# Peterkin

**Published:** 1911-12

**Authors:** 


PETERKIN in an article entitled, "Urologic Facts," *International Journal of Surgery,* September, 1911, emphasizes the fact that the urethral walls are collapsed when at rest but when distended the canal has a capacity of 25 c.c. He questions the value of a 2 drachm syringe in medicating the entire surface of the canal and advocates the use of a douche bag and a glass nozzle.

He also insists that a full bladder is essential when palpating the seminal vesicles. The mechanics of cystocele and its relation to retroversion of the uterus and laceration of the perinaeum are also discussed.

The October number of *The Medical Council* contains an article by Bogart entitled, "One Venereal Menace." He mentions a venereal specialist in a university town who has treated one third of all the students in a single year and his records show that 90 per cent. of the graduates have been under his care in the past.

Another physician who practiced for 30 years in the vicinity of a co-educational school informs him that venereal diseases are rampant among the students of both sexes.

What stronger argument for instruction in sex hygiene, can one find than this.

